# SEOM Clinical Guideline of management of soft-tissue sarcoma (2020)

**DOI:** 10.1007/s12094-020-02534-0

**Published:** 2021-01-06

**Authors:** A. de Juan Ferré, R. Álvarez Álvarez, A. Casado Herráez, J. Cruz Jurado, A. Estival González, J. Martín-Broto, V. Martínez Marín, A. Moreno Vega, A. Sebio García, C. Valverde Morales

**Affiliations:** 1 Hospital Universitario Marqués de Valdecilla, Santander, Spain; 2 Hospital General Universitario Gregorio Marañón, Madrid, Spain; 3 Hospital Universitario Clínico San Carlos, Madrid, Spain; 4 Hospital Universitario de Canarias, Tenerife, Spain; 5 Institut Català d’Oncologia-Badalona, Barcelona, Spain; 6 Hospital Universitario Virgen del Rocío, Sevilla, Spain; 7 Hospital Universitario la Paz, Madrid, Spain; 8 Hospital Universitario Reina Sofía, Córdoba, Spain; 9 Hospital de la Santa Creu I Sant Pau, Barcelona, Spain; 10 Hospital Universitario Vall d’Hebron, Barcelona, Spain

**Keywords:** Sarcoma, Guidelines, Soft-tissue tumors, Uncommon tumors

## Abstract

Soft-tissue sarcomas constitute an uncommon and heterogeneous group of tumors of mesenchymal origin. Diagnosis, treatment, and management should be performed by an expert multidisciplinary team. MRI/CT of the primary tumor and biopsy is mandatory before any treatment. Wide surgical resection with tumor-free tissue margin is the mainstay for localized disease. Radiotherapy is indicated in large, deep, high-grade tumors, or after marginal resection not suitable for re-excision. Perioperative chemotherapy should be discussed for high-risk sarcomas of the extremities and trunk-wall. In the case of oligometastatic disease, patients should be considered for local therapies. First-line treatment with anthracyclines (or in combination with ifosfamide) is the treatment of choice. Other drugs have shown activity in second-line therapy and in specific histological subtypes but options are limited and thus, a clinical trial should always be discussed.

## Introduction

Soft-tissue sarcomas (STS) constitute an uncommon and heterogeneous group of tumors of mesenchymal origin, with an estimated incidence of 5 cases/100,000 inhabitants/year in Europe. They can arise anywhere in the body, but most originate in the extremities, followed by the trunk, retroperitoneum, head and neck, and viscera. Although more common in middle-aged and older adults, they can affect children and young adults. Multidisciplinary management is crucial. STS comprises different histopathological subtypes that share several clinical and pathological features but have some specific characteristics that may impact the treatment.

The main objective of this guideline is to provide updated and clear practical recommendations about the management of STS and to contribute to the improvement of STS patient’s care in Spain. Some subtypes, such as rhabdomyosarcoma, gastrointestinal stromal tumors, extraosseus osteosarcoma, and Ewing’s sarcoma are beyond the focus of this guideline because of their specific and differential management.

## Methodology

These guidelines have been developed by a panel of specialists in medical oncology dedicated to STS in adults. A bibliographic search was performed in PubMed and international guidelines such as NCCN, ESMO/ EURACAN as well as relevant abstracts presented at international meetings were consulted. In a telematic consensus meeting, each section was presented by one expert to the entire group for discussion and consensus. The two coordinating authors were responsible for compiling and homogenizing the different sections. All authors revised and approved the final version of the document. The panel adopted the Infectious Disease Society of America levels of evidence/grades of recommendation [1].

### Diagnosis: imaging, staging, and pathologic diagnosis

As a general principle, both diagnostic and initial treatment should be performed in a coordinated and structured way in expert centers ** (III, A)**, (Table 1) and should take into consideration the patient perspective (Table 2).

**Table 1:** Check list for diagnostic and surgical management

Imaging check list	Pathology check list	Surgery check list
* CT and/or MRI contrast-enhanced before the biopsy	* Core-needle biopsy in reference centre	* Patient’s position
* PET scan is useful to estimate the malignancy grade and response to the neoadjuvant therapy	* Expert validation if diagnosis from not expert center	* Pneumatic cuff (not recommended in sarcoma surgery due to potential dissemination when released). Venous expression is not recommended for the same reason
* A chest spiral CT is mandatory for mediastinal evaluation	* Location, size and depth of tumor	* Asepsis and antisepsis
* Staging by UICC TNM 8th edition	* Primary histological diagnosis by 2020 WHO classification	* Surgical dress preparation
* Brain CT/MRI in alveolar soft part sarcoma, clear cell sarcoma and angiosarcoma	* Histologic grade by FNCLCC grading system (mitotic rate, extent of necrosis and differentiation)	* Surgical approach
* MRI of total spine in myxoid/round cell sarcoma	* Presence or absence of invasion of adjacent structures	* Progressive dissection by anatomical planes
* Bone scan, whole-body MRI are optional	* Surgical report: margins of excision and nodal status, response after neoadjuvant therapy	* Tumour resection
* Additional studies abdominal-pelvic CT/MRI (regional lymph node metastases) in epithelioid sarcoma, angiosarcoma, myxoid/round cell sarcoma, clear cell sarcoma and leiomyosarcoma	* Immunohistochemistry and molecular genetic testing (FISH, RT-PCR or NGS) to detect fusions, translocations and other genetic aberrations in STS	* Anticipated surgical margins (R0-R1-R2) to compare them to those in the pathology report
		* Vascular clipping of the tumour bed macroscopic limits
		* Hemostasis and surgical lavage
		* Surgical closure of tissue planes
		* In case surgical drains, use a drain (passed directly through skin incision) and apply compression dressing and sterile dressing
		* Bandaging and/or plastering

**Table 2:** Patient-centered checklist

Checklist for patients with STS
Patients should be attended at centers belonging to a sarcoma network with a concrete expertise and multidisciplinary team
Children and adolescents should be referred to centers which in addition provide age-specific expertise
A clear treatment plan with objectives and estimated timelines should be discussed with the patient
Psychological and educational support for patients and families is highly recommended
Basal assessment ( especially cardiological with EKG and Echocardio, and endocrine) should be performed and other risk factors should be controlled to reduce toxicity burden of treatments
Oncofertility consultation should be assessed as soon as possible after the diagnosis
Encouraging of physical activity, adapted to patient situation, is highly recommended
Nutritional advice may give a greater sense of well-being and may help to control chemo and radio-therapy side effects
Basal work/ study activity should be assessed and patients should be refered to social workers as needed. Communication with school/university tutors and employers should be encouraged in order to facilitate a realistic plan for reintegration during and after treatment
Getting in touch with other patients and patients’ family through patient associations may reduce isolation feeling and should be offered
Quick activation of palliative care is essential (when indicated)
All patients are entitled to request a second opinion from other oncologist(s)/team without any prejudice

The goal of imaging studies is to establish tumor size, depth, site, resectability, and presence of metastases. Magnetic resonance with contrast is the preferred technic for tumors arising in the limbs, pelvis, and trunk and tomography for retroperitoneal or intraabdominal STS and for staging. The UICC TNM 8th edition [2] is the most spread staging system. Multiple core needle biopsies carried out by an expert after the multidisciplinary discussion is basic for diagnostic. Incisional and excisional biopsy may be considered only in selected cases. Histological diagnosis should be made according to the 2020 WHO Classification [3] and the grade following the FNCLCC-grading system.

CT and/or MRI contrast-enhanced followed by core needle biopsy are the gold standart diagnostic methods. (II, A).

### Treatment localized disease

#### Surgery and radiotherapy (RT)

Surgery is the standard treatment for localized disease. The surgical procedure consists of a wide excision with negative margins (R0) **(II, A)**. The correct negative margins vary depending on the tumour location, histology, grade or preoperative treatment but, generally, at least 1 cm or an intact anatomical barrier is recommended. Reconstructive surgery might help to achieve a R0 surgery. When a wide excision is not possible, amputation/disarticulation is indicated and TNFα and melphalan-based isolated limb perfusion for limb salvage could be considered [4]. Re-operation is mandatory in positive R2 margins and could be considered in R1 when no major morbidity is expected. The biopsy tract should be included in the surgical specimen and the incision should follow the longitudinal axis. Affected lymph nodes should be removed but staging lymphadenectomy not recommended **(III, B)**.

Perioperative RT reduces local recurrences with no impact in survival [5]. RT should be given to high-grade (grade 2–3), > 5 cm and deep lesions **(II, A)**. For tumours without all these high-risk features, RT should be discussed in a multidisciplinary setting. RT is also recommended in tumours resected with close/positive margins and for high-grade local recurrences previously untreated. RT can be administered pre (50 Gy) or post-operatively (50 Gy and optional 10–26 Gy boost depending on the margins) **(II, A)**. Preoperative RT leads to less long-term fibrosis but increases the risk of wound complications. RT can be administered concomitantly with chemotherapy **(III, B)**. Intensity-modulated RT (IMRT) is an option with a better toxicity profile **(III, B)**.

R0 Surgery is the mainstay of treatment **(II, A)**.

#### Adjuvant and neoadjuvant chemotherapy

Perioperative chemotherapy in STS still represents a controversial issue since a meta-analysis with individual data indicated a non-significant absolute difference of 4% favoring chemotherapy administration [6]. In these first-generation randomized trials, a very heterogeneous population was included (different grades, locations, sizes, varied histologies), dose-intensity was low and only 5% of patients received ifosfamide, the second most active drug.

Perioperative chemotherapy with 5 courses of full-dose epirubicin and ifosfamide has demonstrated a significant survival advantage in localized high-risk (G3, > 5 cm and deep) STS of limbs and trunk-wall **(II, A) **[7]. Additionally, a new meta-analysis (although not based on individual data) incorporating 18 comparative trials including a combination of anthracyclines and ifosfamide, resulted in a significant survival advantage (recurrence, distant metastasis and OS) [8]. Neoadjuvant treatment could be preferred over adjuvant since it could add potential prognostic information exploring the interaction between drugs and tumor. Three courses of neoadjuvant full-dose epirubicin and ifosfamide obtained the same result as administering 2 additional courses in the adjuvant setting [5cycles], in high-risk localized STS of limbs and trunk-wall **(II, A)**. Of note, 61% of OS is maintained also over 10 years [9].

There is less convincing evidence for the value of perioperative chemotherapy in STS in other locations and in lower-risk localized patients (i.e. grade 2). Nomograms are increasingly used for the decision-making process so that a risk of death ≥ 40% is used as cut-off for the advice of perioperative chemotherapy.

Perioperative epirrubicin-ifosfamide should be discussed with high-risk patients with tumors arising in limbs and trunk-wall **(II, A)**.

### Treatment of advanced disease

#### Surgery and radiotherapy

Distant metastasis will be developed by 50% of patients diagnosed with STS. Surgery of oligometastatic disease may play a role in well-selected patients, leading to an improvement in OS and 5-year survival rates, according to the retrospective, single-center data (**IV, B**). The presence of a controllable primary tumor, histologic subtype, number of metastatic lesions (oligometastatic disease), low volume disease, and disease-free interval should be considered by the multidisciplinary tumor board. In patients with exclusively metachronous pulmonary metastases, complete resection of lung metastases may attain up to 18–43% long-term survival ** (III, B)** [10]. However, in patients with synchronous lung metastases or short disease-free interval, systemic treatment should be considered. Subsequent surgery could be an option if the benefit is achieved from systemic treatment (**IV, C**). Recurrence of disease after metastasectomy is frequent and there is some evidence to suggest that repeated metastasectomy could be associated with an improvement in the outcome (**IV, C**).

Stereotactic body radiotherapy (SBRT) and stereotactic radiosurgery (SRS) can be used in selected patients who are poor candidates for surgery. SBRT attains excellent tumor control with limited toxicity in patients with lung metastases ** (III, B)** [11]. In subsequent relapses, repeated SBRT may also be considered.

Selected oligometastatic patients may obtain benefit from complete surgical resection or from SBRT/SRS techniques ** (III, B)**.

#### Systemic treatment: First Line

Anthracyclines are still the standard first-line treatment for advanced STS (doxorubicin 75 mg/m^2^ or equivalent) [12] (**I, A**). Although its combination with ifosfamide increased the response rate and progression-free survival in sensitive histological types, it also increased toxicity and it didn´t significantly improve survival in randomized trials (14.3 vs. 12.8 months, HR 0.83) [13] and thus, its reserved for patients who may benefit from tumor reduction for symptom palliation or improving resectability **(I, B**). Ifosfamide at 9–14 g/m^2^ is an alternative for synovial sarcoma or when anthracyclines are contraindicated **(II, A)** and weekly paclitaxel for angiosarcoma (**IIIB**) [14]. Other combination as doxorubicin plus dacarbazine could be considered for patients needing a combination but relatively insensitive to ifosfamide like leiomyosarcoma [15] (V,B).

No drug added to doxorubicin (evofosfamide, palifosfamide, olaratumab) or alternative combination (gemcitabine plus docetaxel) has shown an advantage in overall survival, in recently randomized trials (**I, A**).

Anthracyclines monotherapy is the first-line standard treatment for metastatic patients, not a candidate for local treatment (I,A) and combination therapy could be considered for patients who may benefit from tumor reduction for symptom palliation or improving resectability (I, B).

#### Systemic treatment: second-line chemotherapy and beyond

Second and further lines should be considered in fit and symptomatic patients. For asymptomatic patients, active surveillance may be an option (**IV, C**). Several treatments have been tested in this setting, some of them showing higher responses in specific histotypes. However, none of them has been directly compared. Thus, the decision is based on histology, toxicity profile, and convenience of the scheme administration.

- Ifosfamide: Patients who had not received ifosfamide as a front line could receive it at a dose of 9 g/m^2^ or, in case of progression to standard-dose, at high-dose (≥ 12 g/m^2^) [16] (**III, B**). Synovial sarcoma is especially sensitive to this drug.
- Gemcitabine in combination with docetaxel is more effective in terms of PFS, OS and RR than gemcitabine monotherapy but with increased toxicity [17] (**II, C**). Gemcitabine (1800 mg/m^2^ at 10 mg/m^2^/min) with DTIC (500 mg/m^2^) every 14 days yielded superior OS and PFS when compared to dacarbazine alone with a good toxicity profile [18] (**II, B**), leiomyosarcoma are especially benefits of these combinations.
- Trabectedin (1.5 mg/m^2^ in a 24 h infusion) has been approved in Europe for patients diagnosed with all subtypes of sarcoma after progression, or who are ineligible, for doxorubicin and ifosfamide. A phase III trial, including pre-treated patients with liposarcoma or leiomyosarcoma, demonstrated better PFS with trabectedin over dacarbazine monotherapy (4.2 vs. 1.5 months) [19] (**I, A**). It has shown better responses in myxoid liposarcoma and leiomyosarcoma, and it should be given until progression or intolerable toxicity, although dose and interval modifications may be needed in long-term responders.
- Pazopanib (800 mg daily), a multitargeted tyrosine kinase inhibitor, showed a benefit on PFS versus placebo (4.6 m vs 1.6 m) in a phase III trial in pre-treated patients diagnosed with non-adipocytic sarcoma [20] (**I, A**).
- Eribulin (1.4 mg/m^2^ days 1 and 8/21 days), has been approved for liposarcoma based on a phase III trial comparing eribulin vs dacarbazine after progression to anthracycline. OS was significantly improved (13.5 vs. 11.5 m) in the whole population, without differences in PFS or RR, reaching a 7 month gain in OS in liposarcoma [21]. (**I, A**).

In spite of recent approvals, medical options for metastatic patients are limited and clinical trials, when available, should always be discussed.

Although several treatments have been tested in this setting, none of them has been directly compared. Thus, the decisión of the second line is based on histology, toxicity profile, and convenience of the scheme administration (**IV, A**).

### Therapeutic considerations for specific STS subtypes

#### Retroperitoneal sarcomas

Both retroperitoneal and uterine sarcoma are treated like other sarcomas in the metastasic setting, but there are some special considerations**.**

Retroperitoneal sarcomas (RPS) are characterized by poor prognosis. More than half are high grade and adequate surgical margins are rarely obtained. These patients should be managed by expert surgeons at referral centers with multidisciplinary units and board. *En bloc* resection of the tumor including adjacent organs is the only curative treatment for RPS, negative margins being the main prognostic factor [22] (**III, A**).

Although retrospective studies suggested a possible decrease in local relapses with preoperative RT in resectable tumors, it has not improved survival in a recent randomised clinical trial [23]. Therefore, it is not a standard treatment and should only be considered for selected patients in a multidisciplinary sarcoma tumor board (**II, C**). Intraoperative radiation therapy (IORT) is not standard nor widely available, but a small randomized trial showed its benefit in reducing local recurrence combined with low dose external-beam radiation therapy (EBRT) compared to high dose EBRT. The role of neoadjuvant chemotherapy versus resection alone for RPS is being studied in a randomised clinical trial. It may be considered in case of technically unresectable/borderline resectable RPS, and in chemosensitive histologies (**IV, C**).

Surgery of local recurrences should be considered, especially in cases with a long disease-free interval after previous resection. (**IV B**). En bloc resection performed in high-volume sarcoma centres offers the best chance for long term survival for RPS ** (III, A)**.

#### Uterine sarcomas

Uterine sarcomas (US) includes leiomyosarcomas, high-grade uterine sarcoma, endometrial stromal sarcomas (ESS), as well as other less frequent subtypes like PECOMA and adenosarcoma. Standard local treatment of localized US consists of total hysterectomy with full abdominal cavity exploration. ** (III, A)**. It is not clear whether bilateral salpingo-oophorectomy is always needed. We recommend it for low-grade ESS or tumors expressing ER/PR. (**V, B**) Prophylactic lymphadenectomy is not indicated.

Adjuvant radiotherapy decreases the local relapse rate with no survival benefit and is therefore not routinely considered. It is an option in selected cases with a high relapse risk (I, D). Although adjuvant chemotherapy in uterine LMS is not standard, due to the high risk of systemic relapse, it could be considered in some patients (**IV, C**) [24] Adjuvant hormonal therapy in low-grade ESS is not standard, though it might represent an alternative, given retrospective evidence of increased disease-free interval. (**IV, C**). Hormonal therapy with megestrol acetate, gonadotropin-releasing hormone (GnRH) analogues and aromatase inhibitors is the systemic treatment of advanced low-grade ESS (**IV, B**).

Standard local treatment of localized US is en bloc total hysterectomy ** (III, A)**. Adjuvant RT is not recommended (**I, D**).

#### Desmoid tumor (DT)

DT is a rare monoclonal, fibroblastic proliferation characterized by infiltrative growth and a tendency toward local recurrence but an inability to metastasize. There are two genetic types, CTNNB1 mutations and FAP mutations. The later warrants germline testing and colonoscopy has a more aggressive behavior and is often multifocal.

Active surveillance (clinical & MRI within 1–2 months, then in 3–6 months intervals) by an experienced multidisciplinary team is the best treatment in asymptomatic patients (**III, A**), especially in unfavorable locations: chest wall, head and neck and upper limbs [25] Surgery is considered as the second line, especially in the abdominal wall, provided expected surgical morbidity is limited. Positive microscopic margins can be accepted when function or cosmesis is an issue (**IV, B**). Data for radiotherapy after surgery are limited, but it should be considered when surgery is not an option and medical treatments fail (**IV, B**). There´s no definitive sequence for systemic treatment options. There is randomized data for sorafenib (II, B) and pazopanib (I, B), and phase II evidence for low dose methotrexate-vinblastine and imatinib **(III, B)**. NSAID, antihormonal treatment (**IV, C**), vinorelbine **(III, B)** and liposomal doxorubicin may be options based in retrospective data ** (III, B)**.

Active surveillance by an expert team is the first approach (**III, A**). And use of the less toxic options in the first place is recommended.

#### Solitary fibrous tumor (SFT)

In metastatic or locally advanced malignant SFT, pazopanib is the choice in the first line in typical and malignant subtypes ** (III, B)** [26–27]. Other antiangiogenic agents, such as sunitinib ** (III, B)** or the combination of temozolomide plus bevacizumab, constitute active options (**IV, B**). Chemotherapy in first line, in dedifferentiated SFT, and after the failure of antiangiogenic agents in the rest of subtypes, following the common guidelines for STS which include DTIC-gemcitabine, could be administered but its efficacy is low (**III, C**).

Pazopanib is the first choice in SFT ** (III, B)** except in the dedifferentiated SFT subtype ** (III, B)**.

#### Dermatofibrosarcoma protuberans (DFSP)

DFSP is a cutaneous mesenchymal tumor of intermediate behavior locally aggressive but rarely metastasizing. Surgical excision with wide margins [2–4 cm] is the treatment of localized disease. Mohs surgery can be planned to improve cosmetic results ** (III, B)**. Radiotherapy may be considered if positive margins and unfeasible re-excision. In unresectable, recurrent or metastatic DFSP, imatinib is recommended [28] ** (III, B)**.

Wide resection **(III, B)** and imatinib **(III, B)** if surgery is not possible is the recommended approach for DFSP.

#### Other rare specific subtypes (SS)

In advanced specific subtypes there is evidence of activity of several molecular targeted agents based in preclinical data and small retrospective studies:

- mTor inhibitors and antiangiogenics in PECOMAS and epithelioid hemangioendothelioma (**IV, B**)
- Crizotinib in inflammatory myofibroblastic tumor associated with anaplastic lymphoma kinase (ALK) translocations (**IV, B**)
- Sunitinib ± nivolumab, cediranib in alveolar soft part sarcoma [29] (**IV, C**)
- Sunitinib ± nivolumab in clear cell sarcoma [29] (**IV, C**)
- Tazemetostat in epithelioid sarcoma [30] **(III, B)**
- NTRK sarcomas: entrectinib, larotrectinib [31] ** (III, A)**

Targeted therapies in specific subtypes should be discussed for most patients (IV, B).

### Follow-up

There is no standard follow-up policy. Early detection of local or metastatic recurrence might be potentially curable with surgery, SBRT or other ablative techniques. High-risk extremity STS usually relapses within 2–3 years, mainly to the lungs, although some rare subtypes spread to the lymph nodes (epithelioid, clear cell, synovial sarcoma or rhabdomyosarcoma) and RPS frequently relapse locally, to the liver or peritoneum. Some subtypes like alveolar soft part sarcoma, clear cell sarcoma or extraskeletal chondrosarcoma may relapse even after 10 years and may benefit from longer follow up (**IV, C**). The individual risk of recurrence (size, grade, histological subtype, and site) must be considered to tailor the follow-up strategy (Table 3).

**Table 3:** Follow-up recommendation in STS

		Recommendations	Frequency
Low-grade sarcoma after radical treatment	Local	Physical examination Baseline CT/MRI/US after surgery	First 2–3 years: Every 6 m and then annually
	Distant	Chest X-ray, if M1 nodules, chest CT	Every 6–12 months
Intermediate/high grade sarcoma after radical treatment	Local	Physical examination Baseline CT/MRI/US after surgery	Every 3–4 months
	Distant	Chest X-ray or chest CT	First 2–3 years: Every 3–4 moths 3–5 years: every 6 months after 5 years: annually
Retroperitoneal sarcoma		Abdomino-pelvic CT Chest X-ray, if M1 nodules, chest CT	First 2–3 years: Every 6 months and then annually
Metastatic disease and systemic treatment (outside clinical trial)		Assessment of target lesions (CT, MRI or PET) Individualized follow-up	
Educate patients about self-examination			

**Fig. 1:**
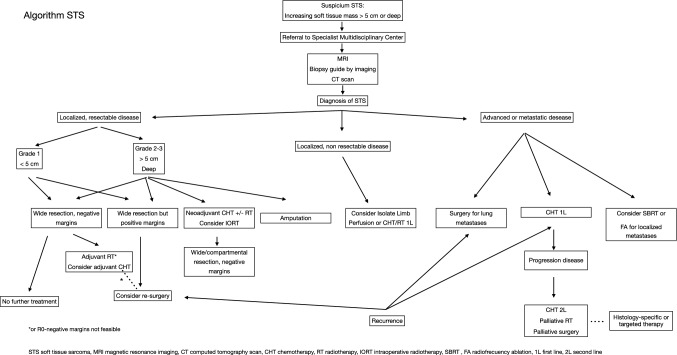
Algorithm for soft tissue sarcoma

Final recommendations and treatment algorithms are summarized in Table 4 and Fig. 1.

**Table 4:** Summary of recommendations

Summary of recommendations
**Patients should be attended at centers belonging to a sarcoma network with a concrete expertise and multidisciplinary team (III,A)**
CT and/or MRI contrast-enhanced followed by core needle biopsy are the gold standart diagnostic methods (**II, A**)
R0 Surgery is the mainstay of treatment **(II, A)**
Perioperative RT is recommended in high-risk tumours **(II, A)**
Perioperative epirubicin-ifosfamide should be discussed with high-risk patients with tumors arising in limbs and trunk-wall **(II, A)**
Selected oligometastatic patients may obtain benefit from complete surgical resection or from SBRT/SRS techniques ** (III, B)**
Anthracyclines monotherapy is the first-line standard treatment for metastatic patients not candidate for local treatment (**I, A**)
Adjuvant combination therapy could be considered for patients who may benefit from tumor reduction for symptom palliation or improving resectability (**I, B**)
Selection of the second line is based on histology, toxicity profile and convenience of the scheme administration. (**IV, A**)
En bloc resection performed in high-volume sarcoma centres offers the best chance for long term survival for retroperitoneal sarcoma ** (III, A)**
Standard local treatment of localized US is en bloc total hysterectomy ** (III, A)**. Adjuvant RT is not recommended (**I, D**)
Active surveillance by an expert team is the first approach for desmoid tumors (III,A) and use of the less toxic options in the first place is recommended
Pazopanib is the first choice in SFT ** (III, B)** except in dedifferentiated SFT subtype ** (III, B)**
Wide resection **(III, B)** and imatinib **(III, B)** if surgery is not possible is the recommended approach for DFSP
Participation in adequatly designed clinical trials, if available, should be discussed with patients (**V, B**)
